# Ecological Interactions in Dinosaur Communities: Influences of Small Offspring and Complex Ontogenetic Life Histories

**DOI:** 10.1371/journal.pone.0077110

**Published:** 2013-10-30

**Authors:** Daryl Codron, Chris Carbone, Marcus Clauss

**Affiliations:** 1 Clinic for Zoo Animals, Exotic Pets and Wildlife, Vetsuisse Faculty, University of Zürich, Zürich, Switzerland; 2 Florisbad Quaternary Research, National Museum, Bloemfontein, South Africa; 3 School of Biological and Conservation Sciences, University of KwaZulu-Natal, Pietermaritzburg, South Africa; 4 Department of Anthropology, University of Colorado at Boulder, Boulder, Colorado, United States of America; 5 Institute of Zoology, Zoological Society of London, London, United Kingdom; Raymond M. Alf Museum of Paleontology, United States of America

## Abstract

Because egg-laying meant that even the largest dinosaurs gave birth to very small offspring, they had to pass through multiple ontogenetic life stages to adulthood. Dinosaurs’ successors as the dominant terrestrial vertebrate life form, the mammals, give birth to live young, and have much larger offspring and less complex ontogenetic histories. The larger number of juveniles in dinosaur as compared to mammal ecosystems represents both a greater diversity of food available to predators, and competitors for similar-sized individuals of sympatric species. Models of population abundances across different-sized species of dinosaurs and mammals, based on simulated ecological life tables, are employed to investigate how differences in predation and competition pressure influenced dinosaur communities. Higher small- to medium-sized prey availability leads to a normal body mass-species richness (*M-S*) distribution of carnivorous dinosaurs (as found in the theropod fossil record), in contrast to the right-skewed *M-S* distribution of carnivorous mammals (as found living members of the order Carnivora). Higher levels of interspecific competition leads to a left-skewed *M-S* distribution in herbivorous dinosaurs (as found in sauropods and ornithopods), in contrast to the normal *M-S* distribution of large herbivorous mammals. Thus, our models suggest that differences in reproductive strategy, and consequently ontogeny, explain observed differences in community structure between dinosaur and mammal faunas. Models also show that the largest dinosaurian predators could have subsisted on similar-sized prey by including younger life stages of the largest herbivore species, but that large predators likely avoided prey much smaller than themselves because, despite predicted higher abundances of smaller than larger-bodied prey, contributions of small prey to biomass intake would be insufficient to satisfy meat requirements. A lack of large carnivores feeding on small prey exists in mammals larger than 21.5 kg, and it seems a similar minimum prey-size threshold could have affected dinosaurs as well.

## Introduction

Modern terrestrial vertebrate systems are dominated by mammals, whereas birds and herpetiles are smaller-bodied and less conspicuous components of our landscapes. This presents a limitation to our understanding of dinosaurian ecology: no contemporary analogue exists from which conclusions can be securely made. One way to overcome this hurdle is to draw from known major differences between mammals and dinosaurs, and to use this information to make inferences about dinosaur ecology and the functioning of Mesozoic land systems. Dinosaurs and mammals differ in multiple aspects of biology, life history, and ecology [1], [2], but it is the difference in reproductive strategies that is likely to have most relevance to arising ecological trends [3]–[6].

Dinosaurs, like their living descendents (birds), and extant herpetiles, were oviparous - numerous eggs and nesting sites have been described from the fossil record, and in some cases these have even been associated with particular taxa [5]–[9]. Mammals, by contrast, are viviparous, and their ancestors were likely giving birth to live young from as early as the Mesozoic [10]. This contrast means that dinosaurs had the higher reproductive output, since oviparous animals can generally produce more offspring (eggs) than the number of live offspring produced by mammals [3], [11]. In terms of life history strategies, species that produce more offspring tend to experience lower survival rates during younger life stages than do species with a lower reproductive output [12]. When survival rates are plotted against age, the patterns that emerge are known as either a type 3 or type B1 survivorship [12]–[14]. In the former, mortality rates level off amongst older individuals such that a negatively concave curve is produced, and in the latter mortality rates become relatively low during the species’ middle years of life, with survivorship decreasing only later in life - the resultant curve is sigmoid in shape. Life tables reconstructed for specific dinosaur taxa directly from the fossil record indicate that they followed type B1 survivorship schedules [13], [14]. Survivorship curves for species with lower reproductive rates (like many mammals) tend to be convex, exhibiting low mortality rates amongst juveniles [15]. Species can achieve this type 1 survivorship by, for example, providing a level of parental care sufficient to ensure that the majority of juveniles escape death by predation, starvation, or disease. Since a species’ survivorship schedule is strongly linked to the growth rate of populations [16], dinosaur populations surely experienced growth and dynamics that were different than those of mammals.

Another outcome of the disparity in reproductive strategies, of equal or potentially even greater significance, is that dinosaurs gave birth to much smaller offspring than do similar-sized mammals [7], [11]. This occurred because, whereas mammals of larger size give birth to offspring of ever-increasing size, dinosaur egg size could not have increased indefinitely. Larger eggs need to be protected by thicker eggshells, but the eggshell cannot be so thick as to prevent sufficient oxygen from diffusing and reaching the growing embryo [9], [17], [18]. Thus, limits to eggshell thickness place limits on maximum egg size, and indeed eggs recovered from the dinosaur fossil record are relatively small compared to the extreme size of the adults, probably not weighing much more than 10 kg (and usually much less than this) in life [7], [9], [11]. As a comparison, offspring of the largest land mammals - the African elephant *Loxodonta africana* and Indian elephant *Elephas maximus* - weigh on average ∼100 kg at birth, respectively [19]. At smaller body sizes, differences in relative offspring size of dinosaurs and mammals were small, but amongst larger size classes the effect is much more notable, with dinosaurs having massive adult- offspring size differences. These dinosaurs would have experienced more complex ontogenetic histories than mammals, with numerous morphological shifts through life [11], [20], [21]. These would have been accompanied by multiple shifts in ecological niches [11], as individuals/species with different morphologies and body masses are often assumed to occupy different niches. Ontogenetic niche shifts would have been even more pronounced in dinosaurs due to limited parental care [22] (young of mammals, which suckle from their mothers, probably have fewer niche shifts through life). Consequently, dinosaur communities must have included a greater diversity of individuals exploiting ecological niches associated with specific body sizes than do mammals, which would have meant a) greater availability of food for predators of the affected size classes, and b) a greater number of individuals competing for shared resources [4], [23]. In the case of the former, younger individuals of the largest dinosaurs would have been available as prey, contrasting with the trophic energy sinks [23] represented by the megaherbivores of modern mammalian ecosystems (whose populations are hardly affected by pressure from predators).

Complex morphological ontogenetic series, and a link between ontogeny and demographic structure, have been described for dinosaur communities [5], [20], [21], but the influence of this structure on the ecology of Mesozoic fauna have hardly been considered in detail. On the other hand, attempts to reconstruct the age/size structure of dinosaur communities directly from the fossil record [13], [14] are questionable because of small sample sizes [24]. Here, we explore size-structured ecological models that reflect the different intensities of key ecological interactions (predation and competition) between dinosaur and mammal communities, to assess how these differences influenced their respective body mass-species richness (*M-S*) distributions, and extinction patterns. We simulate communities comprising size-structured populations across the full range of body size classes expected for both vertebrate groups, and hypothetical life tables for each population based on predicted survivorship schedules (type B1 for dinosaurs, type 1 for mammals). Results are compared with *M-S* distributions from the fossil record (and of extant mammals and birds), to test the hypotheses that 1) middle- and large-sized carnivorous dinosaurs were relatively more diverse than carnivorous mammals [25]–[27] because the former had access to a wider diversity and abundance of prey in this size range [23]; and 2) dinosaurs were poorly represented amongst small-to-middle size class species due to high competition intensity with juveniles from larger species in this range [4]. We also discuss trends in prey size selection that emerge in terms of resource partitioning that occurs amongst different-sized carnivorous dinosaurs in our models.

## Methods

### Vertebrate Body Masses

Body mass data for Mesozoic non-avian dinosaurs, mammals, and birds are from datasets presented in Codron et al. [4] (see references therein for primary literature sources). These include over 120 non-avian dinosaur, 31 bird, and 80 mammal taxa (see Table S1). All data were log_2_-transformed for evaluating *M-S* distributions of each group, as well as for the three major non-avian dinosaur clades separately: Ornithischia, Sauropodomorpha, and Theropoda. The shape of the distributions for each group were evaluated by their skewness, and assessed for normality using the Shapiro Wilks’ test [28]. *M-S* distributions for extant mammals and birds are also presented for comparison. The mammal dataset was extracted from [29], pruned to exclude duplicated species (taking mean body masses for species across continents), the marine Orders Cetacea and Sirenia, and the egg-laying Monotremata. Of the remaining 3501 entries, 214 represent taxa that went extinct by the end of the Pleistocene, and a further 658 are airborne bats (Order Chrioptera) and colugos (Order Dermoptera, *n* = 2), thus the analyses of *M-S* distributions in modern mammals were repeated with both these groups excluded. Further, for comparison with clade-specific trends in dinosaurs, we evaluated *M-S* distributions amongst extant mammalian herbivores and carnivores separately. For the latter, however, we included only mammal groups comprising relatively large taxa, as these were expected to be most comparable with dinosaur communities. Thus, mammalian herbivores are represented by the four living terrestrial ungulate orders (Artiodactyla, Perissodactlya, Proboscidea, and Hyracoidea), and mammalian carnivores by the Order Carnivora. The dataset for bird body masses was taken from [30], including recent updates to that database [31]. We took averages (means) across sexes of the same taxon (including separate means for subspecies), in cases where data for both sexes were provided. The updated data adds numerous new taxa (species and subspecies) to the database, and mass estimates deemed as “better” by the author of the update replaced the earlier estimates. Finally, for taxa where no mean body mass was given, but minimum and maximum masses were, we took the average of the latter. Data for modern mammals and birds are included in Table S1.

### Simulation of Size-Structured Communities

To simulate structure and abundances of dinosaurian and mammalian communities, we specified species (populations) over a variety of size (body mass, *M*, in kg log_2_-transformed) classes, representing the full body mass range described for both groups. For dinosaurs, this range (*i*) extended in log_2_ *M* increments from −9 to 17, and for mammals from −9 to 14, i.e. species ranged in *M* from ∼2 g to 131 and 16 tons, respectively (see Sander et al. [32] for size limits of dinosaur and mammal species). Life tables for each population were constructed, sub-divided by mass classes (*x*) ranging from offspring to adult *M*, again in log_2_ *M* increments. Offspring body masses were estimated by allometric relationships with adult body mass, using a smaller scaling exponent for dinosaurs (0.6) than for mammals (0.9) to incorporate differences in ontogenetic history due to relatively smaller offspring in dinosaurs [3]. These scaling exponents are consistent with available data for extant herpetiles and birds, and for mammals, respectively [3], [18], [33]–[35].

In order to reconstruct survivorship schedules for simulated life tables, we first simulated age-specific survivorships (*g_x_*) using the arbitrary equation
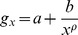
(1)where *a* and *b* are constants greater than and less than zero, respectively, and *x* is the age (body mass) class. Equation 1 produces a negatively concave relationship between *g_x_* and *x* for negative *ρ*, mirroring the hypothetical *g_x_* schedule of populations exhibiting a Type 1 survivorship. For positive values of *ρ*, equation 1 yields a positively concave slope as expected forspecies that exhibit Type 3 survivorship. Because equation 1 produces the desired shape but arbitrary values, *g_x_* schedules had to be standardized across all species in the model. Based on real life tables for 18 mammal and 11 herpetile taxa [36]–[50], which show maximum and minimum *g_x_* values of 0.07 and 0.91, respectively, we standardized our schedules from 0.1 to 0.9. These schedules were then used to estimate mortality rates (*q_x_*, i.e. 1−*g_x_*), and more importantly for life table analyses the standardized survivorships (*l_x_*, i.e. *l_x−_*_1_ *g_x−_*_1_, where *l*_0_ = 1) for each population [12], [16]. Standardized survivorship schedules thus produced convex *l_x_* curves (plotted over *x*) for type 1 survivorships, and concave curves for type 3 survivorships. For dinosaurs, we used a type 1 survivorship, but with *g*_0_ set to the minimum value (i.e. 0.1) to reflect the high mortality rates of the youngest individuals, resulting in the sigmoid curve assumed for type B1 survivorships [13]. Despite concerns about the validity of this type of schedule for dinosaurs [24], we opted to retain the B1 curve since results of an earlier, similar model showed no qualitative differences in final outcomes from a Type 3 survivorship [4] - note that both strategies imply high reproductive output coupled with high infant mortality, reflecting the *r*-strategy predicted for dinosaurs [6]. For mammals, we assumed a Type 1 survivorship, typical for species which practice parental care to a greater degree than most herpetiles, and indeed than what is believed to have occurred in dinosaurs [22].

Fecundity schedules (*m_x_*) of extant mammals and herpetiles are notably asymptotic in shape (when plotted against age); for examples, see [15], [39], [41], [43], [45], [49]. To incorporate this pattern into our simulated life tables, we modeled *m_x_* of each age/size class (*x*) according to the following (arbitrarily-selected) asymptotic equation:
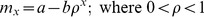
(2)


The minimum breeding stage was set amongst individuals with body masses 10% that of adults for their specific population, although shifting this figure as high as 90% had negligible influences on the end results. Fecundity schedules were then standardized for each population, where maximum *m_x_* scaled negatively (with exponents 0.1) with *M*_adult_ [35].

Finally, we simulated abundances of each age class (*n_x_*), both in terms of numbers available for predation (mortalities in the life tables) and numbers remaining after predation had occurred. Initial abundances for each population were established for the largest size class (*k*) based on negative allometric scaling (exponents −0.75) of body mass with abundance recorded for extant mammals and birds [51]–[53]. Initial abundances for younger age classes were subsequently calculated by multiplying *n* of the largest age class by *l_x_* and dividing by the lowest *l_x_* in the series (i.e.). Abundances of the smallest group (*n*_0_) were added to the number of births, the sum of the fertility schedule (*F_x_*) for each population, where (i.e. the number of individuals in each size class multiplied by their estimated birth rate and survival probability, multiplied by 0.5 assuming only half the population is female). From the series of initial abundances, the numbers eaten by predators were calculated as *n_x_q_x_* (assuming all mortalities are due to predation) and numbers of survivors were calculated as *n_x_*(1−*q_x_*).

### Models of Ecological Interactions

The combined *n_x_q_x_* schedules (assuming these to represent herbivores only, i.e. predation by carnivores on carnivores is omitted here for simplicity) for all populations yielded prey available for carnivores. Our model of predator-prey interactions is based on random encounters between predator and prey individuals of randomly-drawn body masses, similar to an approach used by Carbone et al. [54]. For these simulations, we used the entire mass range as prey, but carnivores ranged in log_2_ *M* from only −9 to 13 (∼8 000 kg) for dinosaurs, and from −9 to 10 (∼1 000 kg) in mammals, since the largest carnivores species that ever existed were somewhat smaller than the largest herbivores. To avoid artificially setting minimum prey sizes taken by a predator, we retained the smallest individuals (log_2_ *M* = −9) for both prey and predators. Two versions of the model were run, incorporating two scenarios. In the first, prey partitioning was assumed *a priori*, so that during any random encounter a successful attack occurred if the predator and prey were of equal body mass. In the second, we assumed niche overlap, with predators consuming any prey individual they encountered that was equal to or smaller than their own mass. Simulations were repeated until the entire prey base was diminished, or results no longer changed with additional simulations - requiring more than 3×10^8^ iterations for each scenario for dinosaurs and mammals, respectively. Ultimately, a matrix of predator-prey mass relationships was produced, from where prey partitioning amongst differently-sized predators could be evaluated, and the *M-S* distributions of predators could be inferred. For the latter, we estimated the number of predator individuals that could be supported by the available prey base from the total mass consumed (kg) by each size class, i.e. the product of numbers of prey eaten and their respective masses. This figure was then divided by the meat requirements for a predator of a particular body mass, which in modern vertebrates typically scales as mass to the exponent 0.75, consistent with allometries of both basal metabolic and field metabolic rates [55], [56]. Meat requirements of herpetiles and mammals likely scale similarly, although the absolute intake (given by the intercept of log-log allometries) may have differed by an order of magnitude depending on whether dinosaurs were ecto- or endothermic [57], [58]. Nonetheless, since ultimately intakes are calculated in relative terms here (i.e. proportions of diet), such physiologically-based differences need not be considered at this stage. In all, our models of predator-prey interactions represent outcomes when only body mass and availability (encounter rates) are considered, but for simplicity we do not include factors such as hunting velocity, energy expenditure, prey defense and predator attack mechanisms, or search areas.

### Incorporating Size-Specific Competition

To incorporate density-dependent competition effects across species, we followed procedures used in a previous version of our models [4]. In brief, only similar-sized individuals (from life tables produced above) “compete”, resulting in mortalities in each size class. The number of deaths were calculated as the total number of individuals of a particular size class, minus the number of individuals in that size class of the population of interest (i.e. competition effects are strictly interspecific), weighted by an arbitrary competition co-efficient (α). In these models, we also evaluate results that incorporate interactions between dinosaurs and mammals as well as those restricted within their respective groups. Finally, following Codron et al. [4], we simulated outcomes of size-specific competition in systems post-dating the non-avian dinosaur extinctions that occurred at the Cretaceous-Tertiary (K-T) boundary. Since these extinctions affected only larger individuals [59], [60], we simply set initial abundances for individuals >25 kg to zero to mimic post K-T conditions.

## Results

### Body Mass-Species Richness Distributions of Dinosaurs, Mammals and Birds

**Figure 1:**
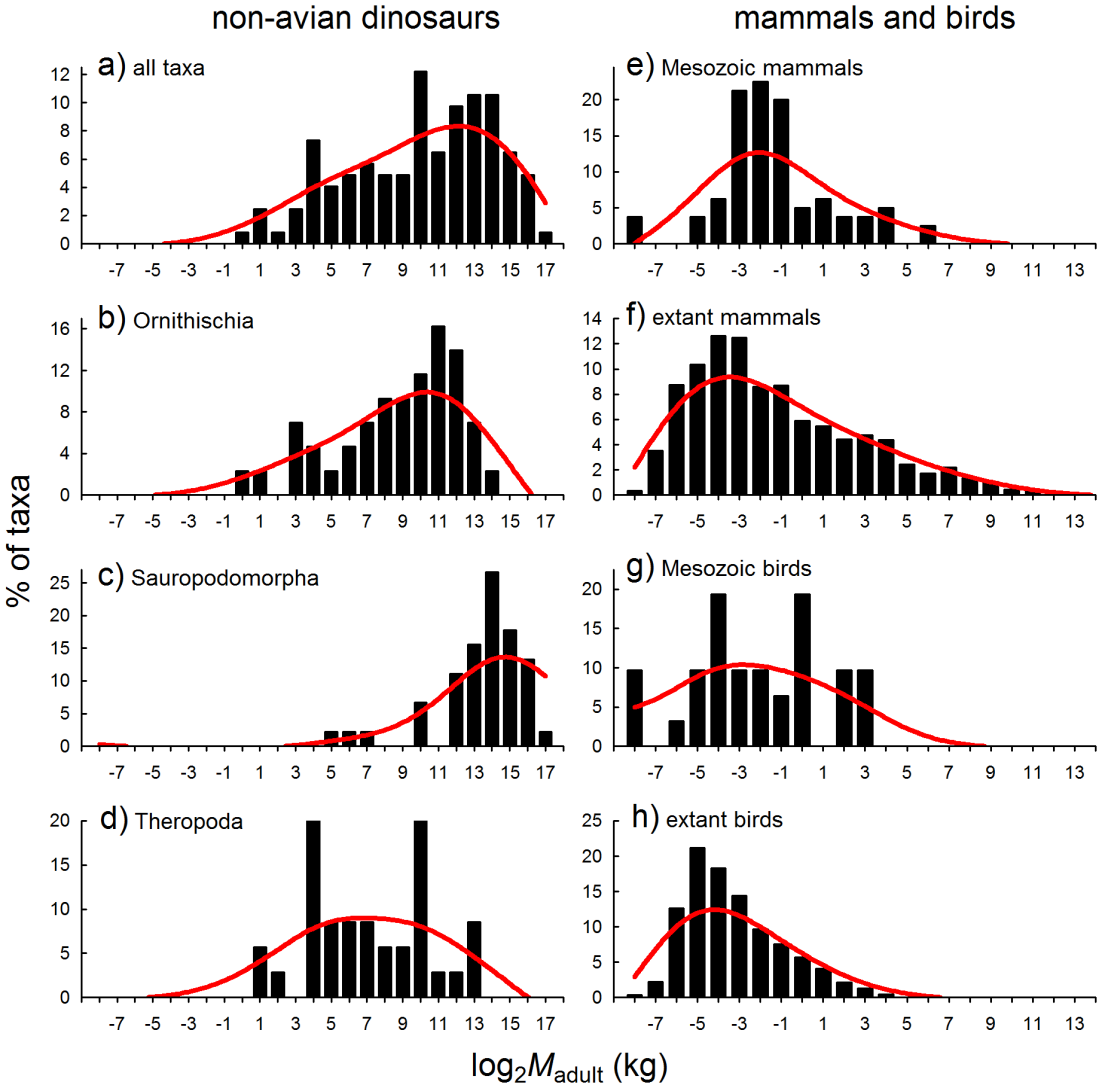
Body mass-species richness (*M-S*) distributions, represented on a log_2_-scale, of extinct (non-avian) dinosaurs, in comparison with distributions of mammals and birds from the Mesozoic and present-day distributions. Data for Mesozoic vertebrates compiled in [4], see references therein for primary sources, and data for extant mammals and birds are from [29]–[31]. Red curves are fitted visually to aid interpretation.

The *M-S* distribution of non-avian dinosaurs in our dataset parallels results from analysis of a much larger dataset [61], and of a spatially-restricted dataset specific to the Dinosaur Park Formation, Alberta [62]. In all three datasets, dinosaurs exhibit a distinct bias against smaller taxa, resulting in left-skewed *M-S* distribution (Fig. 1a). This pattern, however, pertains only to the Ornithischia (Fig. 1b) and Sauropodomorpha (Fig. 1c), whereas the Theropoda - which were evidently better represented amongst smaller and medium-sized classes - display a normal *M-S* distribution, despite peaks at roughly 80 and 1000 kg, respectively (Fig. 1d; SW-*W = *0.952, SW-*p* = 0.127; see Table 1 for a descriptive comparison of distributions and skewness in these groups). Analysis of a dataset comprising nearly 400 non-avian dinosaur taxa revealed a similar difference in *M-S* distributions of ornithsichian and sauropodomorph dinosaurs on the one hand, and theropods on the other [61].

**Table 1:** Descriptive statistics for log_2_ *M*_adult_ (kg) of Mesozoic dinosaur, mammal, and bird taxa, and for living mammals and birds.

Group	*n*	Median	Min	Max	Q25	Q75	Skewness	SW-*W*	SW-*p*
Non-avian dinosaurs									
All taxa	123	10.5	0	17.2	7	13.3	−0.491	0.959	<0.001
Ornithischia	43	9.8	0	14.5	6.7	11.4	−0.826	0.93	0.012
Sauropodomorpha	45	13.7	4.6	17.2	12.7	14.8	−1.599	0.862	<0.001
Theropoda	35	7.3	1.1	12.7	4.3	10	−0.008	0.952	0.127
Mammals									
Mesozoic	80	−3.6	−7.2	4.1	−5	−2.4	1.043	0.921	<0.001
Modern									
Extant	3277	−3.3	−9.2	11.9	−5.3	0.1	0.8	0.939	<0.0001
Incl. recent extinctions	3501	−2.9	−9.2	13.3	−5.2	1.2	0.9	0.928	<0.0001
Excl. airborne groups	2619	−2.3	−9.2	11.9	−4.5	1.3	0.7	0.953	<0.0001
Extant herbivores	223	5.8	1.3	11.9	4.3	7.4	0.2	0.990	0.110
Extant carnivores	258	1.8	−3.3	10.6	0.5	3.8	0.6	0.959	<0.0001
Birds									
Mesozoic	31	−4	−9	1.3	−6.2	−1.6	0.169	0.951	0.168
Extant	9991	−4.8	−9.0	6.8	−6.1	−2.9	0.827	0.999	<0.0001

*n* = number of taxa; SW = Shapiro Wilks’ test for normal distribution.

Modern mammal subgroups: Incl. recent extinctions = data includes species that went extinct in the Late Pleistocene; Excl. airborne groups = data excludes the airborne mammalian orders Chrioptera (bats) and Dermoptera (colugos); carnivores = members of the Order Carnivora; Herbivores = members of the Orders Artiodactyla, Perissodactyla, Proboscidea, and Hyracoidea.

Mammals and birds, by contrast, exhibit more right-skewed *M-S* distributions (normal in the case of Mesozoic birds, but data for this group are limited), both amongst Mesozoic and extant faunas (Figs. 1e–h; Table 1). Similar left-skewed *M-S* distributions have previously been reported for extant mammal and bird assemblages [63]–[65]. An interesting pattern also emerges if data for all oviparous Mesozoic vertebrates are assessed together - because of the small maximum size of Mesozoic birds, the overall Mesozoic terrestrial vertebrate *M-S* distribution is bimodal, and a size gap appears in the size range of several to roughly a thousand kg (Figs. 1a and g; see also Codron et al. [4]). Mammals, which have dominated terrestrial life since the extinction of non-avian dinosaurs 65.6 million years ago, have always had continuous *M-S* distributions [4].

**Figure 2:**
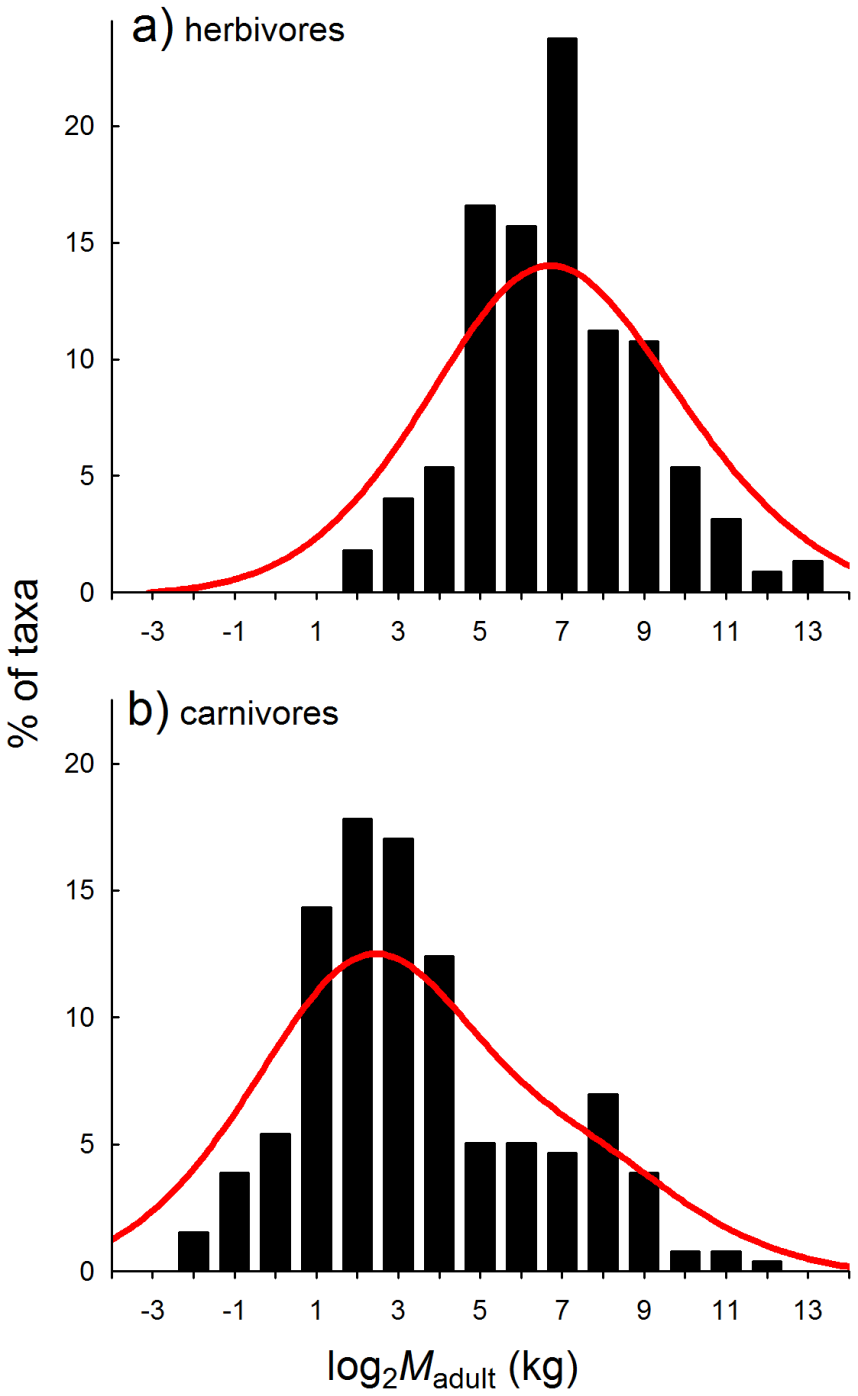
*M-S* distributions of extant mammal herbivores and carnivores. For comparison with *M-S* dinosaur distributions, only larger-bodied groups of mammals were included here, i.e. we omitted data for rodents, insectivores, and other smaller-bodied mammal groups. Thus, herbivores are represented only by the four living ungulate Orders (Artiodactyla, Perissodactyla, Proboscidea, and Hyracoidea), and carnivores by the Order Carnivora. Red curves are fitted visually to aid interpretation.

The difference in *M-S* distributions between ornithischian and sauropod compared with theropod dinosaurs is likely related to differences in trophic positions, since the former comprise largely herbivorous taxa, whereas the latter were primarily carnivores [2], [66]. If this is the case, a further disparity with living mammals can be demonstrated: the large herbivorous land mammals of today (the ungulates) exhibit normal *M-S* distributions across taxa (Fig. 2a; Table 1), whereas the large-bodied carnivores (Order: Carnivora) exhibit strongly right-skewed *M-S* distributions (Fig. 2b). Both groups differ markedly from their Mesozoic dinosaurian counterparts, which had either left-skewed (herbivores) or normal (carnivores) *M-S* distributions, respectively.

### Predator M-S Distributions and Prey Partitioning

**Figure 3:**
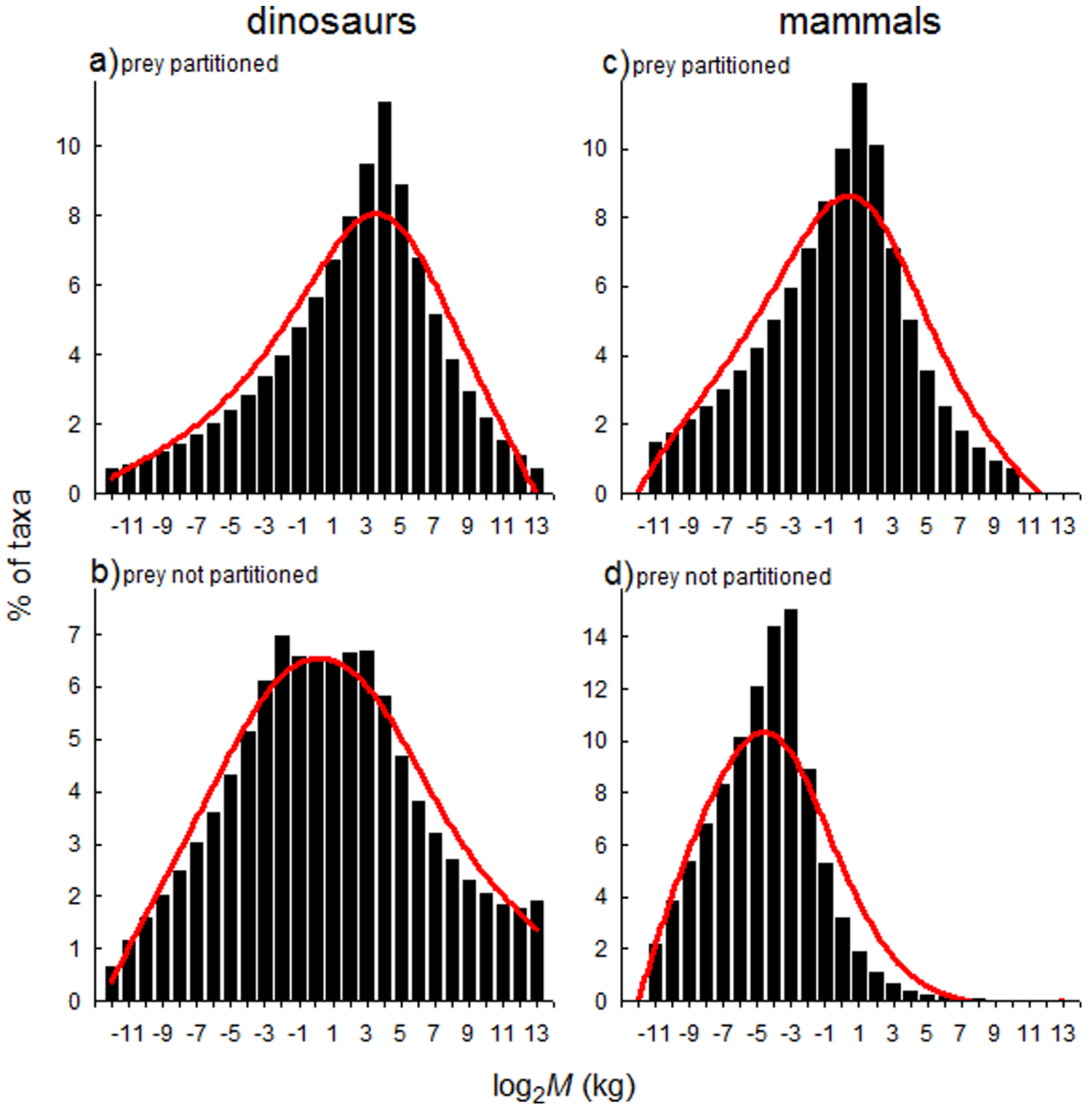
Predicted *M-S* distributions of carnivorous dinosaur and mammal assemblages, based on a model incorporating differences in availability of prey of different body sizes, and the resultant biomass intake (and requirements) by predators. Prey partitioning was assumed by setting prey:predator mass ratios at 1∶1, i.e. each predator is assumed to eat prey of its size only. When prey partitioning was not assumed, predators were allowed to feed on any prey they encountered of their size or smaller. Red curves are fitted visually to aid interpretation.

The *M-S* distribution of dinosaur predators resulting from our model of predator-prey interactions reflects expectations based on prey availability of different sizes, and the intake (biomass) required to support predators of different sizes. The high numbers of intermediate-sized dinosaur prey (i.e. including medium-sized taxa and the younger life stages of larger taxa) presents a richly available food resource for carnivorous dinosaurs. Consequently, the model results in a normal *M-S* distribution of carnivorous dinosaurs, regardless of whether or not prey partitioning is assumed, i.e. whether predators are assumed to consume prey of their size only, or prey of their size and smaller (Figs. 3a and b). This result mirrors the *M-S* distribution of theropod dinosaurs (Fig. 1d), which is normal and contrasts with the strongly left-skewed *M-S* distribution of the primarily herbivorous ornithischian and sauropodomorph groups (Figs. 1b and c). For mammals, a normal *M-S* distribution is also predicted when prey partitioning is assumed (Fig. 3c), but the pattern is distinctly right-skewed when partitioning is not assumed (Fig. 3d). The latter finding is not unlike the *M-S* distribution observed in living members of the Order Carnivora (Fig. 2b).

**Figure 4:**
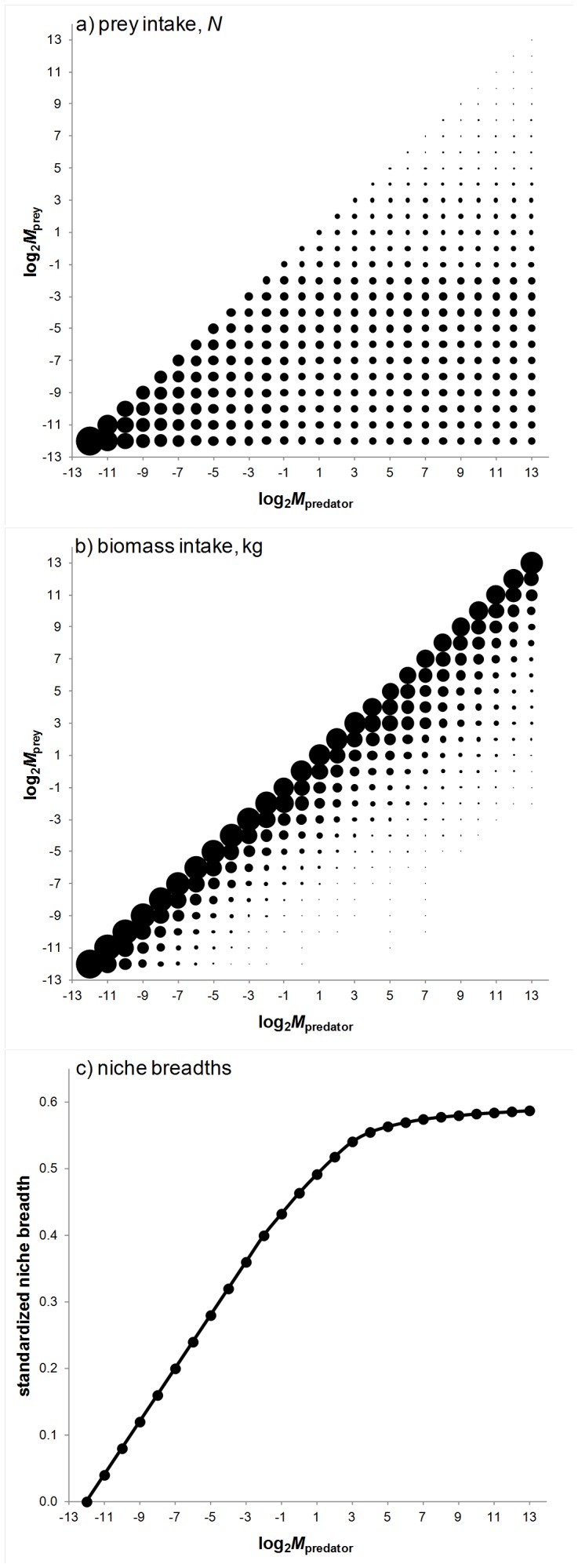
Prey partitioning amongst different-sized predators that arises in models where no prey partitioning was assumed *a priori*. In a) and b) bubbles represent relative contributions of different-sized prey to predator diets, based on numbers or total biomass (kg) consumed, respectively; for c) niche breadths were calculated based relative numbers of prey consumed per size class.

Models in which we assumed no prey partitioning *a priori* yielded results that are informative about the ways in which prey might have been partitioned across carnivorous dinosaurs of different size classes, and in fact how carnivores in general might partition the prey base. In this version of our models, predators were allowed to consume prey up to and including their own mass. The results reflect differences in prey availability across mass classes, such that the smallest predators consume only the smallest prey while larger predators consume an ever-increasing number of prey types (Fig. 4a). Because prey availability (i.e. herbivore density) is negatively correlated with body mass [67], large prey items make up a smaller number of the victims of larger carnivores. However, when relative contributions to a predator’s diet (based on body mass of each item consumed rather than on numbers eaten) are considered, larger prey make up the biggest proportion of the diets of larger predators (Fig. 4b). Actually, above a certain predator mass, proportions of smaller prey items in the total biomass intake of a predator are so small they can be considered negligible. As a result, calculated niche breadths [68] (which are based on relative proportions of different prey items consumed) only increase with predator body mass until about 16 to 32 kg, after which increasing the number of prey items in the diet does not increase dietary diversity (Fig. 4c). The implication is that whereas larger predators can take prey of ever-increasing size, smaller prey items only make substantial contributions to the diets of predators below the 16–32 kg range in our model.

### Size-specific Competition

Effects of size-specific competition on Meoszoic vertebrate communities have been reported previously, based on an earlier version of the models used here [4]. In that study, we predicted that the high degree of size (niche) overlap amongst individuals of small-to-medium size regardless of species resulted in limited niche opportunity for small-to-medium dinosaur species. The net effect is that dinosaur *M-S* distributions would have been bimodal, with a gap in the intermediate size range. Competition from small-bodied mammals would have further reduced niche opportunity for the smallest dinosaur taxa. Thus, if competition between small dinosaurs and mammals was an issue, this would have further reduced the body mass range of the former, leading to their exclusion and/or necessitating adoption of a alternative (i.e. airborne) niches. By contrast, mammal *M-S* distributions would have been continuous except at unrealistically high competition intensities (high α values in the model), but would have been limited to smaller mass classes due to competition pressure from dinosaurs. We predicted that the low species diversity of non-avian dinosaurs amongst the smaller mass range would have prevented the recovery of populations after the K-T extinction events, whereas mammals were able to recover (not having experienced the size gap) and even proliferate into larger mass classes.

Having considered effects of predation on size-structured dinosaur communities in the model versions presented above, it is worth revisiting whether our earlier results of size-specific competitive interactions [4] persist (and also since those effects cannot now be excluded from a detailed analysis of how size structure influenced the ecology of dinosaur communities). In [4], we assumed dinosaurs to have displayed type 3 rather than type B1 survivorships as used here, but we showed in sensitivity analyses that this difference did not influence model outcomes qualitatively. Thus we are only concerned here with the difference in species abundances simulated by the two modelling approaches (here mortalities are also influenced by predation, rather than on mass-abundance scaling alone), and also with the more complex fertility schedules used here (in earlier versions, only the largest individuals within populations produced offspring).

**Figure 5:**
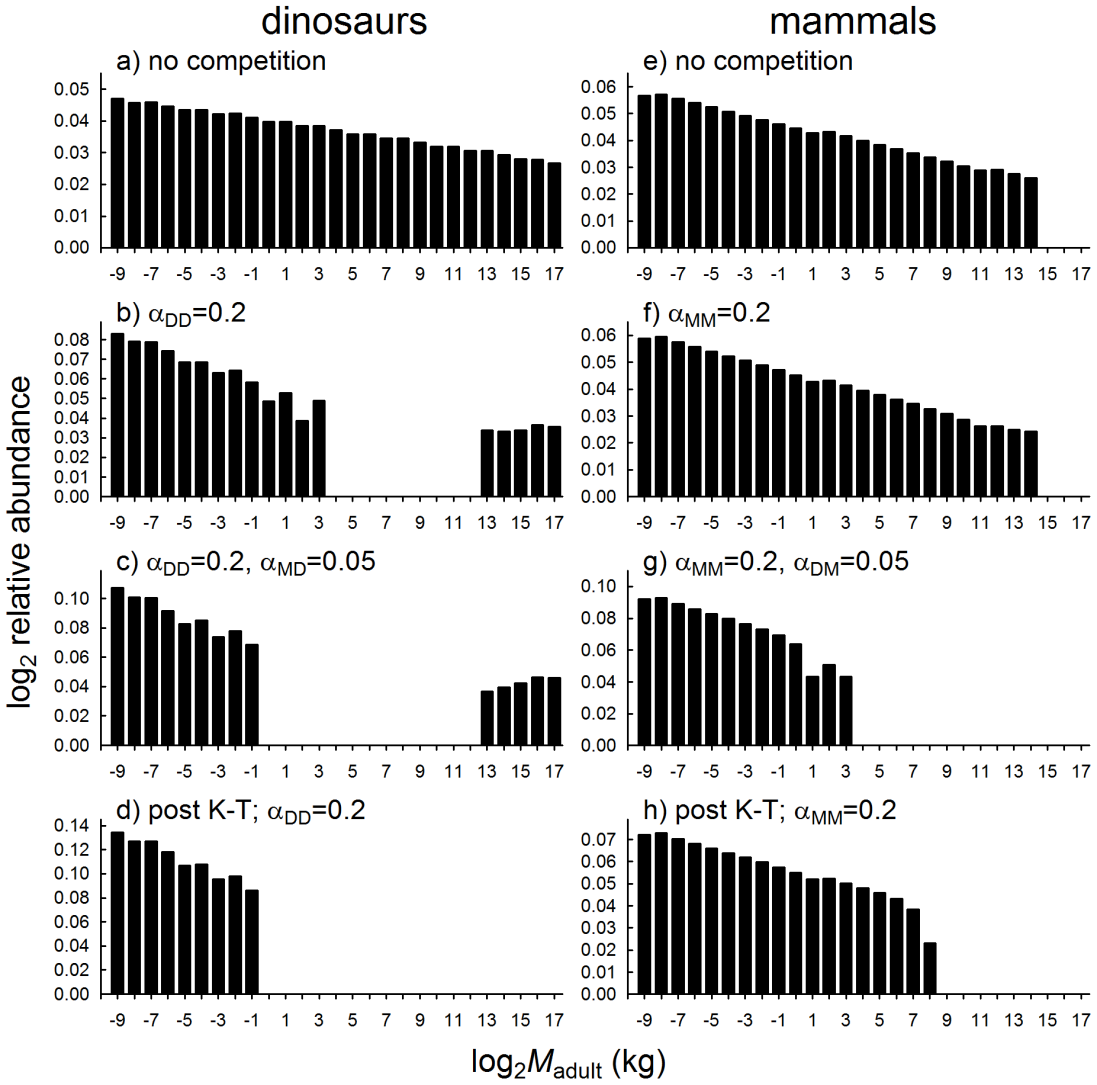
Outcomes of the size-specific competition model, comparing outcomes for *M-S* distributions of dinosaur (with a higher number of size-specific niche overlaps due to their more complex ontogenetic histories) and mammal communities. Competition co-efficients (α) represent the proportion of density-dependent mortalities that occur, due to competition between dinosaurs (subscript DD), between mammals (MM), from mammals on dinosaurs (MD), and from dinosaurs on mammals (DM). Post K-T extinction scenarios were simulated by setting initial conditions to exclude all individuals above 25 kg.

As expected, incorporating size-specific interspecies competition in the present models yielded results that are qualitatively similar to those discussed previously [4], indicating that the high degree of size overlap is a quintessential ecological parameter for dinosaur communities. In the absence of competition, the simulated dinosaur community exhibits a continuous *M-S* distribution (Fig. 5a), but competition-induced mortalities lead to population extinctions in the middle size class range (between several and one thousand kg) resulting in a bimodal *M-S* distribution (Fig. 5b). The lower end of the *M-S* distribution is consistent with minimum and maximum size of Mesozoic birds, whereas few non-avian dinosaur taxa existed in this range (see Figs. 1a and g). In addition, the upper size classes of the small end of the dinosaur *M-S* distribution is further reduced when pressure from competition with other dinosaurs is coupled with competition with similar-sized mammals (Fig. 5c). Finally, results of our simulation of post K-T scenarios (initially excluding all individuals >25 kg) indicate that the body size gap - the explicit outcome of size-specific competition amongst dinosaurs - prevented recovery of populations of larger (non-avian) dinosaur faunas (Fig. 5d).

Effects of size-specific competition are weaker in mammals, due to their less complex ontogenetic histories and lower degrees of size and niche overlaps across species. Our model yields a continuous *M-S* distribution for mammals with and without competition (Figs. 5e and f); size gaps do emerge for mammals, but only at much higher competition intensities than for dinosaurs (e.g. four- or fivefold increases in α). Interestingly, competition with similar-sized dinosaur individuals, including younger life stages of larger dinosaur species, is sufficient enough to result in population extinctions of mammal species above 8 kg (Fig. 5g). Low diversity of mammal species above this size is not unlike what is known about Mesozoic mammals based on the fossil record (see Fig. 1e) - indeed, the largest Mesozoic mammal was only around 30 kg, and this is considered exceptionally large for mammal faunas of the times [69]. In the absence of competition from dinosaurs, post K-T mammals did not suffer this constraint in our model, and populations are able to recover and invade even larger size classes despite initial conditions excluding all individuals above 25 kg (Fig. 5h). Hence, size-specific competition effects, incorporating differences in ontogenetic niche complexities between dinosaurs and mammals, are consistent both with trends observed in the Mesozoic fossil record, and with changes in terrestrial vertebrate diversity after the K-T events.

## Discussion

Dinosaurs differed in numerous ways from mammals, in terms of life history and biology [1], [2]. The respective reproductive strategies of these two groups is a major life history difference, that would have influenced the ecology of both types of communities differently. Notably, no oviparous species since the Mesozoic have reached the massive sizes achieved by dinosaurs, nor even rivalled those of the largest mammals. Yet, even today oviparous and viviparous taxa have disparate life histories, as evident from data collected to construct ecological life tables for mammals and herpetiles [15], [39], [41], [43], [45], [49]. In the case of dinosaurs, an oviparous reproductive strategy coupled with extremely large body size resulted in adult:offspring mass ratios that were substantially higher than those of similar-sized mammals [7]. We hypothesized that this led to a more pronounced and complex ontogenetic series experienced by dinosaurs than mammals, which resulted in a higher frequency of density-dependent ecological interactions in dinosaur- than in mammal-dominated systems.

How ontogenetic niche shifts and resultant changes in the frequency of ecological interactions affect communities is not well understood even in extant systems, but it is likely that population numbers and dynamics would be influenced [70], [71]. Our study focused on resultant changes to community structure, in particular the contrast between extant mammal-dominated and Mesozoic dinosaur-dominated systems. One potential influence at the community level is that more small- to medium-sized prey must have been available to dinosaurian than mammal carnivores. Also, dinosaurs would have experienced more ecological niche shifts through life, as occurs during ontogeny in many species both oviparous and viviparous [70]–[74]. Since similar-sized individuals of a given trophic level often share a similar niche space, the relatively high niche diversity within dinosaur species surely meant more overlaps - and hence more frequent competitive interactions - across species.

### Model Limitations

The size-structured models we used make a number of assumptions about dinosaur life history and ecology which would have influenced our results to some degree. The choice to simulate Type B1 survivorships for dinosaurs (as opposed to Type 1 schedules for mammals) was based on evidence for dinosaur life histories in the fossil record [13], [14]. However, small sample sizes used to construct those life tables may have misleadingly led to inference of concave curves, and only minor adjustments to the data are necessary for convex (Type 3) curves to emerge [24]. Morevoer, many if not most modern herpetiles display Type 3 survivorships [39], [41]–[50], and, given the *r*-life history strategy (i.e. high reproductive output) [1], [6] and low levels of parental care typically expected for non-avian dinosaurs [22], this life history schedule may be more appropriate. Nonetheless, in earlier versions of the model (which focused only on size-specific competition), model outcomes did not differ qualitatively across any type of survivorship schedule, described in Table S1 to [4]. Clearly, the impacts of a more complex size-structure in dinosaur populations than in mammals more significantly influenced community properties than did the shape of species’ survivorship curves. It remains, though, that our models lack variability in life histories across species, and further work is needed to determine what effects - if any - differences in life history of small versus large dinosaurs might have had.

A key assumption of our model is that similar-sized individuals occupy overlapping niche space, and that predators and competitors are strongly influenced by this. While links between body size and niche occupancy should be expected, morphological, physiological, and behavioural constraints could easily dictate an individual’s realized niches and - in theory - lead to niche separation between individuals/species of similar size (recall that these models also do not take differences in carnivore behaviour into account). Our assumption therefore is very general, and makes a broad statement that niche overlaps within body size classes are more frequent than those across body size classes. Thus, our models should not be treated as attempts to quantitatively reconstruct dinosaur communities, but rather to make inferences about broad-scale trends within them.

The assertion that size-specific competition was a major limiting factor in dinosaur-dominated systems is upheld not only by being a logical conclusion deduced from a well-known pattern (the relatively small offsrping of dinosaurs), but also because results presented here are consistent with those presented in an earlier study [4]. The models used in that study lacked effects of predation, and the complexity of breeding schedules used here. Further modification of these approaches will help us to work towards building ever more realistic simulations of past communities and community interactions.

### Comparison to the Fossil Record

The fossil record reveals vastly disparate structures of dinosaur- versus mammal-dominated systems: in the former, *M-S* distributions are bimodal, with a gap in the middle size range between several to around 1 000 kg, whereas *M-S* distributions of the latter are continuous, and have been so throughout the Cenozoic [4]. The size gap in dinosaur-dominated vertebrate systems occurs because of a strong bias towards larger species amongst non-avian dinosaurs [4], [61], and bias towards smaller body size in Mesozoic birds and mammals. Bias towards larger species amongst non-avian dinosaurs means their *M-S* distributions were left-skewed along the mass gradient (whereas mammalian systems are typically right-skewed), although this trend was only consistent amongst herbivorous groups (ornithischians and sauropodomorphs); theropods, having been largely carnivorous, show a more normal pattern. Conversely, the pattern for modern mammalian carnivores is right-skewed, whilst large mammalian herbivores (ungulates) are normally distributed across their body mass range.

The influence of taphonomic effects which could bias *M-S* distributions recovered from the fossil recorded is debatable. While several studies have found no evidence for taphonomic size biases in dinosaur assemblages [8], [61], [75], a recent analysis of a well-constrained assemblage (Dinosaur Park Formation, DPF) suggests that taphonomic effects and researcher bias have resulted in underrepresentation of small-bodied dinosaurs in at least some datasets [62]. However, further analyses of the species accumulation curves (an important source of information for inferring how closely current sampling approximates true diversity) presented in that study reveals that only theropods, not ornithischian dinosaurs, may have been undersampled at DPF (i.e. the curve for ornithischian species richness does reach an asymptote; see also [76]). Hence, even in this spatially-restricted case, the left-skewed *M-S* distribution of the herbivorous group is a consistent trend. Further, the DPF assemblage lacks sauropods, so may in fact underrepresent large-bodied taxa. Whether theropod *M-S* distributions other than normal will emerge from future discoveries is at this stage unclear. Other factors arguing against a major taphonomic effect is that numerous small-bodied mammals and birds have been recovered from a variety of Mesozoic deposits from where small-bodied non-avian dinosaurs are few in number or absent [10]. A recent analysis of a globally-representative dataset found no evidence for taphonomic bias, and in fact reported similar *M-S* distributions as described here [61]. Whatever future discoveries may reveal about Mesozoic dinosaurian and other vertebrate faunas, it seems unlikely that the *M-S* distributions presented here will ever change substantially: for skewness to differ entirely from current predictions, over 95% of non-avian dinosaur taxa still await discovery, all of which would have to be very small [4], [61].

Results of models presented here actually mirror the *M-S* distribution patterns of the dinosaur and mammal fossil records. These results show that left-skewed *M-S* distributions of herbivorous non-avian dinosaurs, and relative scarcity of medium- to small-sized species of this group, could easily have arisen because of size-specific competition for niche space in this mass range. Similarly, the increased availability of medium-sized prey in dinosaur-dominated ecosystems could account for the normal *M-S* distribution so far recorded for theropods, as well as a higher carnivore:herbivore species ratio in dinosaur versus mammal communities [25]–[27]. Thus, our approach offers an ecological explanation for patterns observed in the fossil record, such that we might even expect these patterns rather than predicting that taphonomic effects have taken place.

### Complex Size Structure and the Ecology of Non-Avian Dinosaur Communities

The complex size structure of non-avian dinosaur populations likely influenced carnivores and herbivores in different ways. Whereas here and previously we have predicted a left-skewed *M-S* distribution for non-avian dinosaurs in general [4], data presented here and elsewhere [61] reveal a normal *M-S* distribution amongst the (largely carnivorous) theropods. Our models depict that a high abundance and diversity of prey in the small-medium mass range was available to theropod dinosaurs, because of the numerous younger life stages of very large herbivores that would have been present. This complexity of age/size diversity has also been reported from analyses of dinosaur trackways [5]. Given that carnivores tend to feed on prey at or below their body mass [77], [78], this hypothesized prey diversity could easily explain the higher prevalence of small- and medium-sized carnivorous dinosaurs than observed in the largely herbivorous sauropod and ornithischian clades. A difference from mammal-dominated systems is that megaherbivores did not represent trophic energy sinks [23], as they do in today’s mammalian-dominated systems in which predator pressure on the largest herbivores is small or negligible [79].Similarly, greater diversity and abundance of small- and medium-sized prey in the Mesozoic could have equated to a greater relative (and perhaps absolute) diversity of predators in this size range, explaining the high carnivore:herbivore ratios in these compared with extant mammalian systems (see above). Models converged on this outcome for carnivorous dinosaur assemblages even when prey partitioning was not assumed. Thus, even if the fundamental diet niches of dinosaurian carnivores had overlapped entirely - at least in as much as all had equal access to prey items below their own body size - they still would have been affected differently by prey availability than mammalian carnivores.

When competing for prey in this way, carnivores are likely to partition the prey base due to the interaction between prey availability (which is negatively related to prey size) and energy gain (the mass of the prey). In our models, predators did not consume nearly as many large compared with smaller prey individuals, due to the lower abundances of the former, yet net energy gain (total biomass consumed) made smaller prey items somewhat unprofitable for larger carnivores. Thus, despite the high availability of small prey (in numbers), they contributed little to the overall biomass intake of larger predators. In modern mammals, a switchpoint has been described, around which carnivores smaller than 21.5 kg are represented by taxa that feed primarily at their own body mass and taxa feeding on much smaller prey (including insectivorous species), whereas carnivores larger than 21.5 kg feed only on prey of their own mass [80]. Explanations for this pattern have focused on energetics, a claim supported by models that balance daily net energy expenditure and gain [80], [81]. Our models reveal a similar switchpoint (between ∼16 and 32 kg), which suggests the interaction between prey availability and mass of each meal gained at least partly explains the pattern observed in mammals.

The implication of a prey-size switchpoint is that in dinosaurian carnivore systems - and perhaps amongst vertebrate carnivores in general - there is a high cost associated with feeding on small prey that is related to availability, i.e. above a certain body mass, encounter rates with small prey are insufficient relative to the low energy gain for large predators to forage efficiently. This would force carnivores to focus on larger prey sizes as they themselves increase in size. Nevertheless, given the high productivity of herbivorous dinosaurs in the medium body mass range, most carnivorous dinosaurs would have occupied this feeding niche rather than the high energy requirements needed to catch and subdue very large prey. In other words, by focusing on younger life stages as prey, dinosaurian predators would have been able to ensure that trophic energy was not lost even from populations of the largest herbivore species [23].

Aside from carnivory, our study - consistent with results from an earlier version of these models [4], indicates that size-specific competition was a likely factor driving the bimodal *M-S* distribution of Mesozoic communities, both in terms of limiting niche opportunity for populations of small- and medium-sized non-avian dinosaur populations to flourish, and restricting Mesozoic mammals to small size classes. The combined pressure of competition from mammals and other dinosaurs, if these groups were also competing, could have further restricted niche space available to the smallest dinosaurs. One possible outcome is that very small dinosaurs adopted alternate niches altogether, and the proposed mechanism could thus provide an explanation for the emergence of flight earlier in the Mesozoic. In the absence of large, oviparous taxa having to pass through so many ontogenetic niche stages during growth, size-specific competition has not been as big of an issue for Cenozoic communities.

Oviparity is associated with a higher net reproductive output than viviparity, implying that during the Mesozoic dinosaurs had an advantage over mammals over the various environmental and extinction episodes that occurred [3], [11], [82]. Moreover, their complex ontogenetic histories, including a diversity of niches utilized throughout life, possibly ensured that at least some life stages of dinosaurian populations would have survived through loss of particular habitats during short periods of environmental disturbance. By contrast, loss of only a few habitats during such times would have had far more drastic impacts on mammal populations. However, the K-T events were unique, with events selectively killing individuals above a certain size, probably between 20–25 kg [59], [60]. Our model shows how the lack of species diversity in non-avian dinosaurs at small sizes prevented post K-T recovery of this group. Mammals, and even birds if they were affected, were able to recover because sufficient small-bodied species were present before and after the events. Subsequently, mammals and birds were able to evolve into larger body size classes as well, consistent with the rapid increase of maximum mammal body mass, and increases in avian diversity, from relatively early in the Cenozoic [83], [84].

Dinosaurs are renowned for their large body sizes, and for having had growth rates which were nearly as high as those of endothermic, viviparous mammals [1], [85], [86]. Whether the combined pressure from predation and competition on medium-sized prey populations, and the relative immunity of large adults to these factors, could have been responsible for the evolution of large size and relatively fast growth (for notions linking biology to body size in dinosaurs, see [3], [87]) is an important question for future research, and may shed light on other key aspects of dinosaur evolutionary biology, including the origins of endothermy in them and their living descendents, the birds.

## Supporting Information

Table S1 **Body mass estimates for Mesozoic vertebrates, modern mammals, and modern birds.**
(XLSX)

## References

[1] Sander PM, Christian A, Clauss M, Fechner R, Gee CT, et al (2011) Biology of the sauropod dinosaurs: the evolution of gigantism. Biol Rev (Camb) 86: 117–155. 10.1111/j.1469-185X.2010.00137.x PMC3045712 21251189

[2] Weishampel DB, Dodson P, Osmόlska H (2004) The Dinosauria. Berkeley/Los Angeles, CA: University of California Press.

[3] Werner J, Griebeler EM (2011) Reproductive biology and its impact on body size: comparative analysis of mammalian, avian and dinosaurian reproduction. PLoS ONE 6: e28442. 22194835 10.1371/journal.pone.0028442 PMC3237437

[4] Codron D, Carbone C, Müller DWH, Clauss M (2012) Ontogenetic niche shifts in dinosaurs influenced size, diversity and extinction in terrestrial vertebrates. Biol Lett 8: 620–623. 22513279 10.1098/rsbl.2012.0240 PMC3391484

[5] Lockley MG (1994) Dinosaur ontogeny and population structure: interpretations and speculations based on fossil footprints. In: Carpenter K, Hirsch KF, Horner JR, editors. Dinosaur eggs and babies. Cambridge: Cambridge University Press. 347–354.

[6] Paul GS (1999) Dinosaur reproduction in the fast lane: implications for size, success, and extinction. In: Carpenter K, Hirsch KF, Horner JR, editors. Dinosaur eggs and babies. Cambridge: Cambridge University Press. 244–255.

[7] Sander PM, Peitz C (2008) Upper Cretaceous titanosaur nesting sites and their implications for sauropod dinosaur reproductive biology. Palaeontogr Abt A Palaeozool-Stratigr 284: 69–107.

[8] Carrano MT (2006) Body-size evolution in the Dinosauria. In: Carrano MT, Blob RW, Gaudin T, Wible JR, editors. Amniote paleobiology: perspectives on the evolution of mammals, birds, and reptiles. Chicago: University of Chicago Press. 225–268.

[9] Seymour RS (1979) Dinosaur eggs: gas conductance through the shell, water loss during incubation and clutch size. Paleobiol 5: 1–11.

[10] Benton MJ (2006) Vertebrate palaeontology, third edition. Massachusetts: Blackwell Publishing.

[11] Janis CM, Carrano M (1992) Scaling of reproductive turnover in archosaurs and mammals: why are large terrestrial mammals so rare? Ann Zool Fenn 28: 201–216.

[12] Begon M, Townsend CR, Harper JL (2006) Ecology, from individuals to ecosystems, fourth edition. Oxford: Blackwell Publishing.

[13] Erickson GM, Currie PJ, Inouye BD, Winn AA (2006) Tyrannosaur life tables: An example of nonavian dinosaur population biology. Science 313: 213–217. 16840697 10.1126/science.1125721

[14] Erickson GM, Makovicky PJ, Inouye BD, Zhou C-F, Gao K-Q (2009) A life table for *Psittacosaurus lujiatunensis*: initial insights into ornithischian dinosaur population biology. Anatomical Record: Advances in Integrative Anatomy and Evolutionary Biology 292: 1514–1521. 10.1002/ar.20992 19711482

[15] Heppell SS, Caswell H, Crowder LB (2000) Life histories and elasticity patterns: perturbation analysis for species with minimal demographic data. Ecology 81: 654–665.

[16] Akçakaya HRM, Burgman MA, Ginzburg LR (1999) Applied population ecology, second edition. SunderlandMassachusetts: Sinauer Associates. 285 p.

[17] Ar A, Rahn H, Paganelli CV (1979) The avian egg: mass and strength. Condor 81: 331–337.

[18] Rahn H, Paganelli CV, Ar A (1975) Relation of avian egg weight to body weight. Auk 92: 750–765.

[19] Jones KE, Bielby J, Cardillo M, Fritz SA, O’Dell J, et al (2009) PanTHERIA: a species-level database of life history, ecology, and geography of extant and recently extinct mammals. Ecology 90: 2648 (Ecological Archives E2090–2184)..

[20] Klein N, Sander M (2008) Ontogenetic stages in the long bone histology of sauropod dinosaurs. Paleobiol 34: 247–263.

[21] Chiappe LM (2001) Embryonic skulls of titanosaur sauropod dinosaurs. Science 293: 2444–2446. 11577234 10.1126/science.1063723

[22] Birchard GF, Ruta M, Deeming DC (2013) Evolution of parental incubation behaviour in dinosaurs cannot be inferred from clutch mass in birds. Biol Lett 9. doi:10.1098/rsbl.2013.0036. 10.1098/rsbl.2013.0036 PMC3730617 23676654

[23] Hummel J, Clauss M (2008) Megaherbivores as pacemakers of carnivore diversity and biomass: distributing or sinking trophic energy? Evol Ecol Res 10: 925–930.

[24] Steinsaltz D, Orzack SH (2011) Statistical methods for paleodemography on fossil assemblages having small numbers of specimens: an investigation of dinosaur survival rates. Paleobiol 37: 113–125.

[25] Läng E, Boudad L, Maio L, Samankassou E, Tabouelle J, et al (2013) Unbalanced food web in a Late Cretaceous dinosaur assemblage. Palaeogeogr Palaeoclimatol Palaeoecol 381–382: 26–32.

[26] Farlow JO, Holtz TRJ (2002) The fossil record of predation in dinosaurs. In: Kowalewski M, Kelley PH, editors. The fossil record of predation: Paleontological Society Papers. 251–265.

[27] Horner JR, Goodwin MB, Myhrvold N (2011) Dinosaur census reveals abundant *Tyrannosaurus* and rare ontogenetic stages in the Upper Cretaceous Hell Creek Formation (Maastrichtian), Montana, USA. PLoS ONE 6: e16574. 21347420 10.1371/journal.pone.0016574 PMC3036655

[28] Statsoft_Inc (2007) STATISTICA. Version 8.0 [computer program]. Statsoft Inc. Tulsa, Oklahoma.

[29] Smith FA, Lyons K, Morgan Ernest SK, Jones KE, Kaufman DM, et al (2003) Body mass of Late Quaternary mammals. Ecology 84: 3403 (Ecological Archives: E3084–3094)..

[30] Dunning JB (2007) CRC handbook of avian body masses, second edition: CRC Press.

[31] Dunning JB (2013) Updates to the second edition of the CRC handbook of avian body masses: https://ag.purdue.edu/fnr/Documents/BodyMassesBirds.pdf.

[32] Sander PM, Clauss M (2008) Sauropod gigantism. Science 322: 200–201. 18845734 10.1126/science.1160904

[33] Blueweiss L, Fox H, Kudzma V, Nakashima D, Peters R, et al (1978) Relationships between body size and some life history parameters. Oecologia 37: 257–272. 28309655 10.1007/BF00344996

[34] Cabana G, Frewin A, Peters RH, Randall L (1982) The effect of sexual size dimorphism on variations in reproductive effort of birds and mammals. Am Nat 120: 17–25.

[35] Hendriks AJ, Mulder C (2008) Scaling of offspring number and mass to plant and animal size: model and meta-analysis. Oecologia 155: 705–716. 18196279 10.1007/s00442-007-0952-3 PMC2270366

[36] Armitage KB, Downhower JF (1974) Demography of yellow-bellied marmot populations. Ecology 55: 1233–1245.

[37] Barkalow FS Jr, Hamilton RB, Soots RF Jr (1970) The vital statistics of an unexploited gray squirrel population. J Wildl Manag 34: 489–500.

[38] Barlow J, Boveng P (1991) Modeling age-specific mortality for marine mammal populations. Mar Mamm Sci 7: 50–65.

[39] Berglind S-A (2000) Demography and management of relict sand lizard *Lacerta agilis* populations on the edge of extinction. Ecol Bull 48: 123–142.

[40] Ericsson G, Wallin K, Ball JP, Broberg M (2001) Age-related reproductive effort and senescence in free-ranging moose, *Alces alces* . Ecology 82: 1613–1620.

[41] Ortega-Rubio A, Halffter G, Barbault R (2000) Bunch grass lizard, *Sceloporus scalaris*, population dynamics at La Michilia Biosphere Reserve, Mexico. Herpetol J 10: 33–39.

[42] Parker WS (1974) Demography of the fence lizard, *Sceloporus undulatus*, in Northern Mississippi. Copeia 1994: 136–152.

[43] Galán P (1999) Demography and population dynamics of the lacertid lizard *Podarcis bocagei* in north-west Spain. J Zool 249.

[44] Abts ML (1985) The life history strategy of the Saxicolous desert lizard, *Sauromalus obesus*: Portland State University.

[45] Congdon JD, Dunham AE, van Loben Sels RC (1994) Demographics of common snapping turtles (*Chelydra serpentina*): implications for conservation and management of long-lived organisms. Am Zool 34: 397–408.

[46] Punzo F (2007) Life history, demography, diet and habitat associations in the southwestern earless lizard, *Cophosaurus texanus scitulus* from northern and southern limits of its geographical range. Amphibia-Reptilia 28: 65–76.

[47] Vinegar MB (1975) Demography of the striped plateau lizard, *Sceloporus virgatus* . Ecology 56: 172–182.

[48] Tinkle DW, Dunham AE (1983) Demography of the tree lizard, *Urosaurs ornatus*, in central Arizona. Copeia 1983: 585–598.

[49] Van Devender RW (1982) Comparative demography of the lizard *Basiliscus basiliscus* . Herpetologica 38: 189–208.

[50] Gadsden H, Estrada-Rodríguez JL (2008) Demography of the Yarrow’s spiny lizard, *Sceloporus jarrovii*, from the central Chihuahuan desert. West N Am Nat 68: 46–57.

[51] Damuth J (1981) Home range, home range overlap, and species energy use among herbivorous mammals. Biol J Linn Soc 15: 185–193.

[52] Damuth J (2007) A macroevolutionary explanation for energy equivalence in the scaling of body size and population density. Am Nat 169: 621–631. 17427133 10.1086/513495

[53] Blackburn TM, Gates S, Lawton JH, Greenwood JJD (1994) Relations between body size, abundance and taxonomy of birds wintering in Britain and Ireland. Philos Trans R Soc Lond B 343: 135–144.

[54] Carbone C, Turvey SJ, Bielby J (2011) Intra-guild competition and its implications for one of the biggest terrestrial predators, *Tyrannosaurus rex* . Proc R Soc Lond B Biol Sci 278: 2682–2690. 10.1098/rspb.2010.2497 PMC3136829 21270037

[55] McNab BK (2008) An analysis of the factors that influence the level and scaling of mammalian BMR. Comp Biochem Physiol A 151: 5–28. 10.1016/j.cbpa.2008.05.008 18617429

[56] Nagy KA, Girard IA, Brown TK (1999) Energetics of free-ranging mammals, reptiles, and birds. Annu Rev Nutr 19: 247–277. 10448524 10.1146/annurev.nutr.19.1.247

[57] Brett-Surman MK, Farlow JO (1997) Some irrelevant thoughts about dinosaur metabolic physiology: jurisphagous food consumption rates of *Tyrannosaurus rex*. In: Farlow JO, Brett-Surman MK, editors. The complete dinosaur. Bloomington: Indiana University Press. 350–351.

[58] Clauss M, Steuer P, Müller DWH, Codron D, Hummel J (2013) Herbivory and body size: allometries of diet quality and gastrointestinal physiology, and implications for herbivore ecology and dinosaur gigantism. PLoS ONE (this issue). 10.1371/journal.pone.0068714 PMC3812987 24204552

[59] Robertson DS, McKenna MC, Toon OB, Hope S, Lillegraven JA (2004) Survival in the first hours of the Cenozoic. GSA Bullettin 116: 760–768.

[60] Archibald JD (1996) Dinosaur extinction and the end of an era. New York: Columbia University Press.

[61] O’Gorman EJ, Hone DWE (2013) Body size distribution of the dinosaurs. PLoS ONE 7: e51925. 10.1371/journal.pone.0051925 PMC3526529 23284818

[62] Brown CM, Evans DC, Campione NE, O’Brien LJ, Eberth DA (2012) Evidence for taphonomic size bias in the Dinosaur Park Formation (Campanian, Alberta), a model Mesozoic terrestrial alluvial-paralic system. Palaeogeogr Palaeoclimatol Palaeoecol 372: 108–122.

[63] Smith FA, Brown JH, Haskell JP, Lyons SK, Alroy J, et al (2004) Similarity of mammalian body size across the taxonomic hierarchy and across space and time. Am Nat 163: 672–691. 15122486 10.1086/382898

[64] Brown JH, Maurer BA (1989) Macroecology - the division of food and space among species on the continents. Science 243: 1145–1150. 17799895 10.1126/science.243.4895.1145

[65] Blackburn TM, Gaston KJ (1994) The distribution of body sizes of the world’s bird species. Oikos 70: 127–130.

[66] Zanno LE, Makovicky PJ (2011) Herbivorous ecomorphology and specialization patterns in theropod dinosaur evolution. Proc Natl Acad Sci USA 108: 232–237. 21173263 10.1073/pnas.1011924108 PMC3017133

[67] Damuth J (1981) Population density and body size in mammals. Nature 290: 699–700.

[68] Levins R (1968) Evolution in changing environments. Princeton, NJ: Princeton University Press.

[69] Hu Y, Meng J, Wang Y, Li C (2005) Large Mesozoic mammals fed on young dinosaurs. Nature 433: 149–152. 15650737 10.1038/nature03102

[70] Wissinger SA (1992) Niche overlap and the potential for competition and intraguild predation between size-structured populations. Ecology 73: 1431–1444.

[71] Werner EE, Gilliam JF (1984) The ontogenetic niche and species interactions in size-structured populations. Annu Rev Ecol Syst 15: 393–425.

[72] Radloff FGT, Hobson KA, Leslie AJ (2012) Characterising ontogenetic niche shifts in Nile crocodile using stable isotope (δ^13^C, δ^15^N) analyses of scute keratin. Isotopes Environ Health Stud 48: 439–456. 22462522 10.1080/10256016.2012.667808

[73] Woolley L-A, Page B, Slotow R (2011) Foraging strategy within African elephant family units: Why body size matters. Biotropica 43: 489–495.

[74] Codron J, Kirkman K, Duffy KJ, Sponheimer M, Lee-Thorp JA, et al (2013) Stable isotope turnover and variability in tail hairs of captive and free-ranging African elephants (*Loxodonta africana*) reveal dietary niche differences within populations. Can J Zool 91: 124–134.

[75] Foster JR (2007) Jurassic West. The dinosaurs of the Morrison Formation and their World. Bloomington: Indiana University Press.

[76] Codron D, Carbone C, Müller DWH, Clauss M (2012) Ecological modelling, size distributions and taphonomic size bias in dinosaur faunas: reply to Brown *et al*. Biol Lett doi: 10.1098/rsbl.2012.0922. 10.1098/rsbl.2012.0922 PMC3565508 23310990

[77] Brose U, Jonsson T, Berlow EL, Warren P, Banasek-Richter C, et al (2006) Consumer-resource body-size relationships in natural food webs. Ecology 87: 2411–2417. 17089649 10.1890/0012-9658(2006)87[2411:cbrinf]2.0.co;2

[78] Troost TA, Kooi BW, Dieckmann U (2008) Joint evolution of predator body size and prey-size preference. Evol Ecol 22: 771–799.

[79] Sinclair ARE, Mduma S, Brashares JS (2003) Patterns of predation in a diverse predator–prey system. Nature 425: 288–290. 13679915 10.1038/nature01934

[80] Carbone C, Mace GM, Roberts SC, Macdonald DW (1999) Energetic constraints on the diet of terrestrial carnivores. Nature 402: 286–288. 10580498 10.1038/46266

[81] Carbone C, Teacher A, Rowcliffe JM (2007) The costs of carnivory. PLoS Biology 5: e22. 17227145 10.1371/journal.pbio.0050022 PMC1769424

[82] Werner J (2010) Die Reproduktion von Dinosauriern, speziell der Sauropoden und deren Bedeutung für ihren Gigantismus: Johannes Gutenberg-Universität Mainz.

[83] Smith FA, Boyer AG, Brown JH, Costa DP, Dayan T, et al (2010) The evolution of maximum body size of terrestrial mammals. Science 330: 1216–1219. 21109666 10.1126/science.1194830

[84] Feduccia A (2003) ‘Big bang’ for tertiary birds? Trends Ecol Evol 18: 172–176.

[85] Clarke A (2013) Dinosaur energetics: setting the bounds on feasible physiologies and ecologies. Am Nat 182: 283–297. 23933721 10.1086/671259

[86] Werner J, Griebeler E (2013) Case curves on growth rate and body mass revised: dinosaurs had rather growth rates like ectotherms than endotherms. PLoS ONE (this issue).

[87] Sookias RB, Benson RBJ, Butler RJ (2012) Biology, not environment, drives major patterns in maximum tetrapod body size through time. Biol Lett 8: 674–677. 22513278 10.1098/rsbl.2012.0060 PMC3391459

